# A non-linear model of hydrogen production by *Caldicellulosiruptor saccharolyticus* for diauxic-like consumption of lignocellulosic sugar mixtures

**DOI:** 10.1186/s13068-018-1171-3

**Published:** 2018-06-22

**Authors:** Johanna Björkmalm, Eoin Byrne, Ed W. J. van Niel, Karin Willquist

**Affiliations:** 1 Department of Energy and Circular Economy, RISE Research Institutes of Sweden, PO Box 857, 501 15 Borås, Sweden; 2 Division of Applied Microbiology, Lund University, PO Box 124, 221 00 Lund, Sweden

**Keywords:** *Caldicellulosiruptor saccharolyticus*, Hydrogen, Kinetic growth model, Glucose uptake, Xylose uptake, Diauxic

## Abstract

**Background:**

*Caldicellulosiruptor saccharolyticus* is an attractive hydrogen producer suitable for growth on various lignocellulosic substrates. The aim of this study was to quantify uptake of pentose and hexose monosaccharides in an industrial substrate and to present a kinetic growth model of *C. saccharolyticus* that includes sugar uptake on defined and industrial media. The model is based on Monod and Hill kinetics extended with gas-to-liquid mass transfer and a cybernetic approach to describe diauxic-like growth.

**Results:**

Mathematical expressions were developed to describe hydrogen production by *C. saccharolyticus* consuming glucose, xylose, and arabinose. The model parameters were calibrated against batch fermentation data. The experimental data included four different cases: glucose, xylose, sugar mixture, and wheat straw hydrolysate (WSH) fermentations. The fermentations were performed without yeast extract. The substrate uptake rate of *C. saccharolyticus* on single sugar-defined media was higher on glucose compared to xylose. In contrast, in the defined sugar mixture and WSH, the pentoses were consumed faster than glucose. Subsequently, the cultures entered a lag phase when all pentoses were consumed after which glucose uptake rate increased. This phenomenon suggested a diauxic-like behavior as was deduced from the successive appearance of two peaks in the hydrogen and carbon dioxide productivity. The observation could be described with a modified diauxic model including a second enzyme system with a higher affinity for glucose being expressed when pentose saccharides are consumed. This behavior was more pronounced when WSH was used as substrate.

**Conclusions:**

The previously observed co-consumption of glucose and pentoses with a preference for the latter was herein confirmed. However, once all pentoses were consumed, *C. saccharolyticus* most probably expressed another uptake system to account for the observed increased glucose uptake rate. This phenomenon could be quantitatively captured in a kinetic model of the entire diauxic-like growth process. Moreover, the observation indicates a regulation system that has fundamental research relevance, since pentose and glucose uptake in *C. saccharolyticus* has only been described with ABC transporters, whereas previously reported diauxic growth phenomena have been correlated mainly to PTS systems for sugar uptake.

## Background

The need for renewable energy is ever increasing to tackle the major challenges of global warming, energy demand, and limited resources. According to statistics published by the International Energy Agency [1], just over 86% of the Total Primary Energy Supply (TPES) in 2014 was produced from fossil resources, leaving a modest 14% originating from renewable energy sources. When putting these numbers in relation with the adopted Paris Agreement in 2015, targeting to keep the global average temperature increase below the 2 °C above pre-industrial levels [2], it is evident that actions need to be taken. There are, however, positive trends in that the supply of renewable energy sources has grown faster, with an average annual rate of 2.0% since 1990, compared to the growth of the world TPES of 1.8% [1].

Hydrogen has the potential of becoming an important renewable energy carrier. Currently, hydrogen is widely used as a reducing agent in the chemical and food industry. However, using hydrogen as an energy carrier in sustainable applications is of great interest due to its potentially high efficiency of conversion to usable power, its low emissions of pollutants and high energy density [3]. Up to 96% of the world’s hydrogen production is fossil based, i.e., natural gas, oil, and coal [4]. A sustainable alternative to the conventional methods for producing hydrogen is by biological methods, i.e., biohydrogen. There are four major categories in which production of biological hydrogen can be classified, namely: photofermentation of organic compounds by photosynthetic bacteria, biophotolysis of water using algae and cyanobacteria, bioelectrohydrogenesis, and fermentative hydrogen production, so-called dark fermentation, from organic wastes or energy crops [5, 6]. The latter is the focus of this study, where various sugars present in, e.g., agricultural waste like wheat straw, can be fermented by microorganisms for hydrogen production. This also addresses the challenge of converting lignocellulosic biomass to renewable energy.

Lignocellulosic biomass has been previously described as “the most abundant organic component of the biosphere” with an annual production of 1–5·10^13^ kg and, therefore, is an attractive substrate for biofuel production [7]. Lignocellulosic biomass primarily consists of cellulose (40–60% CDW), hemicellulose (20–40%), and lignin (10–25%) [8]. Cellulose and hemicellulose can be enzymatically hydrolyzed into smaller sugar molecules.

The thermophilic microorganism *Caldicellulosiruptor saccharolyticus* is able to produce hydrogen from lignocellulosic biomass through dark fermentation and has previously shown the potential of producing hydrogen close to the maximum theoretical yield of 4 mol hydrogen per mol hexose [9–11]. *C*. *saccharolyticus* is cellulolytic and can utilize a broad range of di- and monosaccharides for hydrogen production [12]. Van de Werken et al. [13] showed that *C. saccharolyticus* coferments glucose and xylose as it lacks catabolite repression. VanFossen et al. [14] revealed that although *C. saccharolyticus* co-utilizes different sugars, it has a preference for some sugars over others. Xylose was discussed as a preferred sugar over glucose and is, therefore, utilized by the microorganism to a greater extent than glucose. However, the substrate uptake kinetics was not determined and a yeast extract (YE)-supplemented medium was used [13].

By developing a mathematical model for a biological process, it is possible to describe past and predict future performances as well as gaining a deeper understanding of the physiological mechanism behind the process. The aim of this study is to present a model that describes the growth of *C*. *saccharolyticus* on lignocellulosic sugar mixtures and how the uptake rate changes when the sugars are used simultaneously or individually. Similar kinds of models have been developed [15, 16]; however, these models focus on single sugar uptake. The proposed model here builds on the one presented by Ljunggren et al. [15] by adding the consumption rates for each individual sugar in the sugar mixtures. Monod [17] first described the phenomenon of diauxic growth, where a microorganism is exposed to two substrates and first consumes the substrate that supports the most efficient growth rate. Several models have been developed in this area [18, 19] describing how to capture the subsequent uptake of sugars when multiple sugars are present. This phenomenon can be modeled using a cybernetic approach to whether a particular enzyme, needed for a specific sugar to be metabolized, is upregulated or not.

This paper describes the development of a substrate-based uptake model using Monod-type kinetics including biomass growth, product formation, liquid-to-gas mass transfer, and enzyme synthesis with Hill kinetics, with *C. saccharolyticus* as model organism. The model presented in this paper takes into consideration the usage of different sugars, including hexoses, i.e., glucose, and pentoses, i.e., xylose and arabinose. The model describes the different sugar uptakes individually, exemplifying the rate at which each sugar is consumed when *C. saccharolyticus* grows on the sugar mixtures and on the individual sugars, respectively.

## Methods

### Strains and cultivation medium

*Caldicellulosiruptor saccharolyticus* DSM 8903 was obtained from the Deutsche Sammlung von Mikroorganismen und Zellkulturen (Braunschweig, Germany). Sub-cultivations were conducted in 250 mL serum flasks with 50 mL modified DSM 640 media [20]. The carbon source of each cultivation corresponded to that of the subsequent fermentor cultivation. The 1000× vitamin solution and modified SL-10 solution were prepared according to [20] and [21], respectively.

All bioreactor experiments used a modified DSM 640 medium with the exclusion of yeast extract according to Willquist and van Niel [20]. To quantify the kinetics of xylose and glucose uptake and the effect of when the sugars were mixed in pure and industrial medium, the growth and hydrogen production was monitored in four different cases, where the total sugar concentration in the medium was fixed to 10 g/L. Cultivations were performed using 10 g/L glucose (Case 1), 10 g/L xylose (Case 2), a sugar mixture (Case 3), and wheat straw hydrolysate (Case 4). In Case 4, a 9% solution of wheat straw hydrolysate was used corresponding to approximately 10 g/L total sugars. In Case 3, the sugar mixture contained pure sugars with the same concentration as the wheat straw hydrolysate (6.75 g/L glucose, 3.06 g/L xylose, and 0.173 g/L arabinose). The total sugar concentrations at the start of the fermentation included the sugar added as described above and the additional sugar added from the inoculum, which varied slightly in the different conditions. The starting sugar concentration was, therefore, as follows: Case 1, 12.11 ± 0.09 g/L glucose; Case 2, 10.96 ± 0.20 g/L xylose; Case 3, 8.69 ± 0.12 g/L glucose, 3.38 ± 0.19 g/L xylose, and 0.38 ± 0.01 g/L arabinose; Case 4, 7.31 ± 0.07 g/L glucose, 3.36 ± 0.06 g/L xylose, and 0.34 ± 0.00 g/L arabinose.

### Fermentor setup

Batch cultivations were performed in a jacketed, 3-L fermentor equipped with ADI 1025 Bio-Console and ADI 1010 Bio-Controller (Applikon, Schiedam, The Netherlands). A working volume of 1 L was used for cultivations and the pH was maintained at optimal conditions 6.5 ± 0.1 at 70 °C by automatic titration with 4 M NaOH. The temperature was thermostatically kept at 70 ± 1 °C. Stirring was maintained at 250 rpm and nitrogen was sparged through the medium at a rate of 6 L/h. Sparging was initiated 4 h after inoculation and was continued throughout the cultivation. A condenser cooled with water at 4 °C was utilized to prevent evaporation of the medium. Samples were collected at regular time intervals for monitoring of the optical density. The supernatant from each culture was collected and stored at − 20 °C for further quantification of various sugars and organic acids. Gas samples were collected from the fermentor’s headspace to quantify H_2_ and CO_2_. The sugar mixture and wheat straw hydrolysate experiments were done in triplicate. The individual sugar fermentations were done in biological duplicate.

A defined medium was autoclaved in each fermentor, while anoxic solutions of cysteine HCl·H_2_O (1 g/L), MgCl_2_·6H_2_O (0.4 g/L), and carbon source(s) were prepared separately and were added to the fermentor before inoculation. Just after inoculation, the fermentor was closed for 4 h to allow buildup of CO_2_ as previously described [20] necessary to initiate growth.

### Analytical methods

Optical density was determined using an Ultraspec 2100 pro spectrophotometer (Amersham Biosciences) at 620 nm. Sugars, organic acids, hydroxymethyl furfural (HMF), and furfural were detected using HPLC (Waters, Milford, MA, USA). For the quantification of organic acids, an HPLC equipped with an Aminex HPX-87H ion-exchange column (Bio-Rad, Hercules, USA) at 60 °C and 5 mM H_2_SO_4_ as mobile phase was used at a flow rate of 0.6 mL/min. Glucose, xylose, and arabinose quantification was conducted using an HPLC with a Shodex SP-0810 Column (Shodex, Japan) with water as a mobile phase at a flow rate of 0.6 mL/min. CO_2_ and H_2_ were quantified with a dual channel Micro-GC (CP-4900; Varian, Micro-gas chromatography, Middelburg, The Netherlands), as previously described [21].

### Mathematical model description

The model developed for *C. saccharolyticus* in this study takes into account the kinetics of biomass growth, consumption of glucose, xylose and arabinose, and formation of the products acetate, hydrogen, and carbon dioxide. Furthermore, the model includes liquid-to-gas mass transfer of hydrogen and carbon dioxide as well as the equilibrium between carbon dioxide, bicarbonate (HCO_3_^−^) and carbonate (CO_3_^2−^). The model is developed on a cmol basis. The formation of lactate was excluded to reduce the complexity of the model, as it constituted to less than 5% of the total product in the sugar mixture fermentations. In addition, inhibition due to high aqueous H_2_ concentration and high osmolarity was not included in the model to reduce the number of unknown parameters. This is motivated by the fact that the focus of this study is mainly on the consumption behavior of *C. saccharolyticus* on the different sugars.

The model is constructed with a similar nomenclature and setup as in the anaerobic digestion model no 1 (ADM1) described by Batstone et al. [22] and was implemented in MATLAB R2015b (Mathworks, USA). The following biochemical degradation reactions are the basis for the model (Eqs. 1, 2).

Biomass formation from sugar [23]:

1 $$ {\text{Sugar }} \mathop \to \limits^{{\rho_{1} }}  Y_{X}  {\text{CH}}_{1.62} {\text{O}}_{0.46} {\text{N}}_{0.23} {\text{S}}_{0.0052} {\text{P}}_{0.0071} . $$

Reaction 1 is not balanced, since there were elements in the fermentation medium that were not included in the model, i.e., cysteine. The value of the yield factor *Y*_*X*_ is calculated from the data of the batch fermentations. It is assumed that nitrogen, sulfur, and phosphorus are in excess in the media and, therefore, are not included as separate entities in the mathematical model.

Sugar degradation to product formation by *C. saccharolyticus* in cmol:

2 $$ {\text{CH}}_{ 2} {\text{O}} + {\raise0.7ex\hbox{$1$} \!\mathord{\left/ {\vphantom {1 3}}\right.\kern-0pt} \!\lower0.7ex\hbox{$3$}}{\text{H}}_{ 2} {\text{O}} \mathop \to \limits^{{\rho_{2} }}  {\raise0.7ex\hbox{${ 2}$} \!\mathord{\left/ {\vphantom {{ 2} 3}}\right.\kern-0pt} \!\lower0.7ex\hbox{$3$}}{\text{CH}}_{ 2} {\text{O}} + {\raise0.7ex\hbox{$1$} \!\mathord{\left/ {\vphantom {1 3}}\right.\kern-0pt} \!\lower0.7ex\hbox{$3$}}{\text{CO}}_{ 2} + {\raise0.7ex\hbox{$2$} \!\mathord{\left/ {\vphantom {2 3}}\right.\kern-0pt} \!\lower0.7ex\hbox{$3$}}{\text{H}}_{ 2} . $$

#### Model inputs and initial conditions

The model requires a range of input variables. The lag time was determined by calculating the intersection point between the lag phase and the exponential phase when taking the natural logarithm of the biomass concentration over time, as illustrated by Swinnen et al. [24]. Since the lag phase is dependent on the culture status before the fermentation, which was not addressed in this study, it was excluded from the experimental data when the latter were compared to model data and for initial input values for the model. The start values of the unknown state variables are listed in Table 1. The constants used in the model are presented in Table 2.

**Table 1:** Start data of the unknown state variables in the model

State variable	Description	Case 1 Glucose fermentation	Case 2 Xylose fermentation	Case 3 Sugar mix fermentation	Case 4 Wheat straw hydrolysate fermentation	Unit
Glu	Glucose concentration	0.40	–	0.28	0.26	cmol/L
Xyl	Xylose concentration	–	0.36	0.10	0.11	cmol/L
Ara	Arabinose concentration	–	–	0.012	0.014	cmol/L
*X* (Biomass)	Biomass concentration	0.0013	0.00071	0.0016	0.0058	cmol/L
Ac	Acetate concentration	0.0012	0	0.0039	0.021	cmol/L
H_2,aq_	H_2_ concentration (liquid phase)	0	0	0	0	M
CO_2,aq_	CO_2_ concentration (liquid phase)	0	0	0	0	cmol/L
CO_2,sol_	Concentration of all CO_2_ ionic species (HCO_3_− and CO_3_^2^−)	0	0	0	0	cmol/L
H_2,g_	H_2_ concentration (gas phase)	0	0	0	0	M
CO_2,g_	CO_2_ concentration (gas phase)	0	0	0	0	cmol/L
*E*2	Enzyme concentration	–	–	1e−7	1e−7	cmol/L

**Table 2:** Constants used in the model

Constant	Value	Unit	Refs
*V*_liq_, liquid volume	1	L	
*V*_gas_, gas volume	0.05	L	[15]
pH	6.5	–	
*k*_AB_, acid base rate constant^a^	1e4	–	
*T*, temperature	343.15	K	
*R*, ideal gas constant	0.08206	L atm/K/mol	
${\text{KH}}_{{{\text{H}}_{2} }}$, Henry’s constant H_2_	7.4e−9	mol/L/Pa	
${\text{KH}}_{{{\text{CO}}_{2} }}$, Henry’s constant CO_2_	2.7e−7	mol/L/Pa	
$k_{\text{L}} a_{{{\text{CO}}_{2} }}$, volumetric mass transfer coefficient for carbon dioxide	5.85·(N_2_/6)^0.46^	h^−1^	[15]
p*K*_1_, dissociation constant of reaction forming bicarbonate	6.3	–	
p*K*_2_, dissociation constant of reaction forming carbonate	10.25	–	
*β*, enzyme decay rate	0.05	h^−1^	[18]
N_2_, stripping rate	6	L/h	

^a^ The acid–base reaction is considered to be in equilibrium at all times, which means that the reactions have infinitely fast reaction rates

#### Mass balances for biomass growth, substrate consumption, and product formation in the liquid phase

The stoichiometric relationships and mass balances of the reactants and products present in the model are displayed in Table 3. The model is supplemented with an enzyme, *E*2, and cybernetic variables *v* and *u* as in [18], where the former controls the activity of the enzyme and the latter is the fractional allocation of a critical resource for the synthesis of the enzyme. We hypothesize that initially, there is a first enzyme system present aiding the subsequent uptake of both hexose and pentose sugars, but with a preference for the pentoses (phase I). This transporter is only available as long as pentoses are present. After depletion of the pentoses, a second enzyme system, *E*2, is synthesized allowing for uptake of the remaining hexose sugars by a second transporter (phase II). For the sake of convenience, we simplify the enzyme system, consisting of multiple proteins, using the word enzyme and using this abstraction also in the kinetic model.

**Table 3:** Description of the model setup including mass balances for the sugars (glucose, xylose, and arabinose), enzyme *E*2, biomass, acetate, aqueous hydrogen, and aqueous carbon dioxide

Phase I	Phase II	Process↓								
		Glu	Xyl	Ara	Ac	H_2,aq_	CO_2,aq_	*E*2	*X*	Rate (*ρ*, cmol/L/h)
Glu		− 1			(1 − *Y*_*x*_)·*Y*_ac_	(1 − *Y*_*x*_)·$Y_{{{\text{H}}_{2} }}$	(1 − *Y*_*x*_)·$Y_{{{\text{CO}}_{2} }}$		*Y*_*x*_	$\rho_{\text{Glu}} = k_{\text{m}} \cdot \frac{\text{Glu}}{{{\text{Glu}} + K_{\text{s,glu}} }} \cdot X \cdot v_{1}$
	Glu	− 1			(1 − *Y*_*x*_)·*Y*_ac_	(1 − *Y*_*x*_)·$Y_{{{\text{H}}_{2} }}$	(1 − *Y*_x_)·$Y_{{{\text{CO}}_{2} }}$	− 1·*E*2	*Y*_*x*_	$\rho_{{{\text{Glu}},2}} = k_{{{\text{m}},2}} \cdot E2 \cdot \frac{\text{Glu}}{{{\text{Glu}} + K_{{{\text{s,glu}},2}} }} \cdot X \cdot v_{2}$
Xyl			− 1		(1 − *Y*_*x*_)·*Y*_ac_	(1 − *Y*_*x*_)·$Y_{{{\text{H}}_{2} }}$	(1 − *Y*_*x*_)·$Y_{{{\text{CO}}_{2} }}$		*Y*_*x*_	$\rho_{\text{Xyl}} = k_{\text{m}} \cdot \frac{\text{Xyl}}{{{\text{Xyl}} + K_{\text{s,xyl}} }} \cdot X \cdot v_{1}$
Ara				− 1	(1 − *Y*_*x*_)·*Y*_ac_	(1 − *Y*_*x*_) · $Y_{{{\text{H}}_{2} }}$	(1 − *Y*_*x*_)·$Y_{{{\text{CO}}_{2} }}$		*Y*_*x*_	$\rho_{\text{Ara}} = k_{\text{m}} \cdot \frac{\text{Ara}}{{{\text{Ara}} + K_{\text{s,ara}} }} \cdot X \cdot v_{1}$
	Enzyme, *E*2 (synthesis)							1		$\rho_{E} = \alpha \cdot \frac{{{\text{Glu}}^{n} }}{{{\text{Glu}}^{n} + K_{{{\text{s}},E2}}^{n} }} \cdot X \cdot u$
	Enzyme, *E*2 (decay)							− 1		$\rho_{{{\text{dec,}}E2}} = \beta \cdot E2$
Biomass (decay)	Biomass (decay)								− 1	$\rho_{{{\text{dec,}}X}} = r_{\text{cd}} \cdot X$
										$v_{1} = \frac{{\rho_{\text{Xyl}} }}{{\hbox{max} \;\left( {\rho_{\text{Xyl}} ;\;\rho_{{{\text{Glu,}}2}} } \right)}}$ $v_{2} = \frac{{\rho_{{{\text{Glu}},2}} }}{{\hbox{max} \;\left( {\rho_{\text{Xyl}} ;\;\rho_{{{\text{Glu}},2}} } \right)}}$
										$$ u = \frac{{\rho_{{{\text{Glu}},2}} }}{{{\text{sum }}\left( {\rho_{\text{Xyl}} ;\;\rho_{{{\text{Glu,}}2}} } \right)}} $$

At the bottom of the table, the cybernetic variables *v* and *u* are described

The mass balance for the biomass, *X*, is dependent on the rate of substrate consumption *ρ*, with Monod-type kinetics, and on the biomass decay rate, which is described with first-order kinetics, where *r*_cd_ (h^−1^) is the cell death rate and *Y*_*x*_ (cmol/cmol) is the yield of biomass from total sugar (Table 3). A second glucose rate equation ($\rho_{\text{Glu, 2}}$) is added to describe the diauxic-like growth appearance in the sugar mixture. The rate of the glucose consumption, when the pentose sugars are depleted, is dependent on enzyme *E*2. The rate of the enzyme synthesis, *ρ*_*E*_, is based on Hill kinetics, as in [19], the decay rate of the enzyme is first-order kinetics, and the third term, − 1·*E*2·$\rho_{\text{Glu, 2}} ,$ represents the dilution of the specific enzyme level as is described with kinetics similar to Hill, i.e., *E*2^2^. The parameters *k*_m_ and *k*_m,2_ (h^−1^) are the maximal uptake rates in phase I and phase II, respectively, and *K*_s,glu_, *K*_s,glu,2_, *K*_s,xyl_, *K*_s,ara,_ and *K*_*s*,*E*2_ (cmol/L) are the affinity constants for the uptake of glucose, xylose, arabinose, and synthesis of enzyme *E*2, respectively. Finally, *α* is the enzyme synthesis rate (h^−1^) and *β* is the enzyme decay rate (h^−1^).

Acetate, hydrogen, and carbon dioxide are produced in the liquid phase. *Y*_ac_ (cmol/cmol), $Y_{{{\text{H}}_{ 2} }}$ (mol/cmol) and $Y_{{{\text{CO}}_{ 2} }}$ (cmol/cmol) represent the conversion yields of acetate, hydrogen, and carbon dioxide, respectively, from both hexose and pentose sugars. The conversion yields were fitted with experimental data from the batch fermentations. *Y*_*X*_ was determined by the slope of the curve: total sugar vs biomass; here, only phase I was considered. *Y*_ac_ and $Y_{{{\text{CO}}_{ 2} }}$ were determined by first taking the slope of the curves, total sugar vs acetate, and total sugar vs carbon dioxide, and then, the actual yields were calculated according to the following equation:

3 $$ Y_{\text{Ac}} = \frac{{Y_{\text{Ac, curve slope}} }}{{1 - Y_{X} }} . $$

When $Y_{{{\text{H}}_{ 2} }}$ was calculated the same way as in Eq. 3, it gave a too high conversion yield. To obtain a more accurate yield, the effects of liquid-to-gas mass transport were considered and $Y_{{{\text{H}}_{ 2} }}$ was instead determined as follows:

4 $$ Y_{{{\text{H}}_{ 2} }} = \frac{{{\text{H}}_{{ 2 , {\text{end}}}} - {\text{H}}_{{ 2 , {\text{start}}}} }}{{{\text{Tot sugar}}_{\text{start}} - {\text{Tot sugar}}_{\text{end}} }}. $$

#### Acid–base reactions

The acid–base reaction considered in the model is that of carbon dioxide, bicarbonate, and carbonate formation. $\rho_{{{\text{AB,CO}}_{ 2} }}$ in Table 4 describes the rate of formation of bicarbonate and carbonate.

**Table 4:** Kinetic rate equation for the acid–base reaction

	Process↓		
	CO_2,sol_	CO_2,aq_	Rate (*ρ*_*t,j*_, cmol/L/h)
CO_2_ acid–base	1	− 1	$\rho_{{{\text{AB,CO}}_{ 2} }} = k_{\text{AB}} \cdot ({\text{CO}}_{{ 2 , {\text{aq}}}} \cdot \left( {\frac{{10^{{ - {\text{p}}K_{1} }} }}{{10^{{ - {\text{pH}}}} }} + 10^{{ - {\text{p}}K_{1} }} \cdot \frac{{10^{{ - {\text{p}}K_{2} }} }}{{\left( {10^{{ - {\text{pH}}}} } \right)^{2} }}} \right) - {\text{CO}}_{{ 2 , {\text{sol}}}}$

CO_2,sol_ is the sum of the ionic species, ${\text{HCO}}_{3}^{ - }$ and CO_3_^2−^ and Eq. 5 gives the differential equation for CO_2,sol_:

5 $$ \frac{{d{\text{CO}}_{{ 2 , {\text{sol}}}} }}{{{\text{d}}t}} = \rho_{{{\text{AB,CO}}_{ 2} }} . $$

#### Liquid-to-gas mass transfer and mass balances for product formation

Hydrogen and carbon dioxide are produced in the liquid phase and then transferred to the gas phase via liquid-to-gas mass transport. $\rho_{{t,{\text{H}}_{2} }}$ describes the mass transfer rate of hydrogen and $\rho_{{t,{\text{CO}}_{2} }}$ is the mass transfer rate of carbon dioxide (Table 5). $p_{{{\text{gas,H}}_{ 2} }}$ and $p_{{{\text{gas,CO}}_{ 2} }}$ (in atm then converted to Pa) are the partial pressures of H_2_ and CO_2_, respectively.

**Table 5:** Liquid-to-gas mass transfer processes

	Process↓				
	H_2,g_	CO_2,g_	H_2,aq_	CO_2,aq_	Rate (*ρ*_*t,j*_, cmol/L/h)
H_2_ transfer	1		− 1		$\rho_{{t,{\text{H}}_{ 2} }} = k_{L} a_{{{\text{H}}_{ 2} }} \cdot ({\text{H}}_{{2 , {\text{aq}}}} - p_{{{\text{gas,H}}_{ 2} }} \cdot {\text{KH}}_{{{\text{H}}_{ 2} }} )$
CO_2_ transfer		1		− 1	$\rho_{{t,{\text{CO}}_{ 2} }} = k_{\text{L}} a_{{{\text{CO}}_{ 2} }} \cdot ({\text{CO}}_{{ 2 , {\text{aq}}}} - p_{{{\text{gas,CO}}_{ 2} }} \cdot {\text{KH}}_{{{\text{CO}}_{ 2} }} )$

The expression for the mass balances describing the gaseous products can be described as in Eqs. 6, 7, where $q_{\text{gas}}$ (L/h) is the total gas flow, and *V*_liq_ and *V*_gas_ (L) are the liquid and the gas volumes, respectively:

6 $$ \frac{{{\text{dH}}_{{ 2 , {\text{g}}}} }}{{{\text{d}}t}} = \frac{{V_{\text{liq}} }}{{V_{\text{gas}} }}\cdot\rho_{{t,{\text{H}}_{ 2} }} + \left( { - {\text{H}}_{{ 2 , {\text{g}}}} \cdot \frac{{q_{\text{gas}} }}{{V_{\text{gas}} }} } \right) $$

7 $$ \frac{{{\text{dCO}}_{{2,{\text{g}}}} }}{{{\text{d}}t}} = \frac{{V_{\text{liq}} }}{{V_{\text{gas}} }}\cdot\rho_{{t , {\text{CO}}_{2} }} + \left( { - {\text{CO}}_{{ 2 , {\text{g}}}} \cdot \frac{{q_{\text{gas}} }}{{V_{\text{gas}} }} } \right). $$

### Sensitivity analysis

A sensitivity analysis can identify parameters that have great effect on the model output. The sensitivity analysis was done based on the OFAT approach, i.e., one-factor-at-at-time [25]. The chosen parameter was altered with a factor *δ*, as described in [26], to see the effect on the different state variable output result, as in the following equation:

8 $$ \varGamma_{i,j} = \frac{{\left( {y_{i} \left( {\theta_{j} } \right) - y_{i} \left( {\theta_{j} + \delta \cdot \theta_{j} } \right)} \right)/y_{i} (\theta_{j} )}}{\delta }, $$

where *Γ*_*i,j*_ is the sensitivity of state variable *i* with respect to model parameter *j* in each timepoint of the Matlab simulation. Furthermore, *y*_*i*_(*θ*_*j*_) is the value of state variable *i* in regard to parameter *j* and $y_{i} \left( {\theta_{j} + \delta \cdot \theta_{j} } \right)$ is the value of state variable *i* when parameter *j* has been altered with a factor *δ*. The parameters that were included in the sensitivity analysis were *k*_m_, *k*_m,2_, *K*_s,glu_, *K*_s,glu,2_, *K*_s,xyl_, *K*_s,ara_, *K*_s,E2_, *α*, *n*, *r*_cd,_ and *k*_L_a_H2_ and the state variables that were considered were Glu, Xyl, Ara, Ac, *X,* and H_2_. The presented sensitivity data of one parameter in regards to a specific state variable were calculated as the average of *Γ*_*i,j*_.

### Model calibration

To get a better fit to the experimental data, the model parameters were calibrated using the knowledge that was revealed in the sensitivity analysis. This was done with the function *lsqcurvefit* in MATLAB which uses a least square method to find the right parameter value for a non-linear curve fitting by seeking to find coefficients *x* that solve the problem in the following equation:

9 $$ \mathop {\text{min} }\limits_{x} \left\| F\left( {x,x{\text{data}}} \right) - y{\text{data}}\right\|_{2}^{2} = \mathop {\text{min} }\limits_{x} \mathop \sum \limits_{i} \left( {F\left( {x,x{\text{data}}_{i} } \right) - y{\text{data}}_{i} } \right)^{2} $$

given the input data *x*data and the observed output *y*data, where *x*data and *y*data are matrices or vectors and *F(x*,*x*data*)* is a matrix-valued or vector-valued function of the same size as *y*data.

The *lsqcurvefit* function starts at *x*0 and finds coefficient, i.e., parameter *x*, to best fit the non-linear function fun*(x*,*x*data*)* to the data *y*data:

10 $$ x = lsqcurvefit({\text{fun}},x0,x{\text{data}},y{\text{data}}). $$

The uncertainties of the calibrated parameters were assessed by calculating the confidence interval. This was done with the function *nlparci* in MATLAB which computes the 95% confidence intervals for the non-linear least square parameters estimated.

## Results and discussion

### Growth profiles on the various sugars

**Fig. 1:**
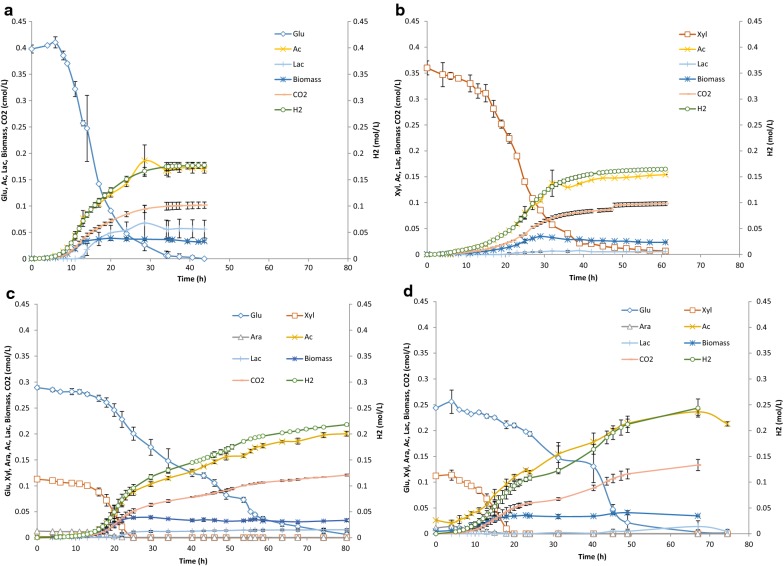
Fermentation profiles of Cases 1–4: **a** glucose experiment, **b** xylose experiment, **c** sugar mixture experiment, and **d** wheat straw hydrolysate experiments. The error bars indicate the standard deviation. *Glu* glucose, *Xyl* xylose, *Ara* arabinose, *Ac* acetate, *Lac* lactate, *X* biomass

The growth profiles of the single sugar experiments (glucose; Case 1 and xylose; Case 2), sugar mixture experiments (Case 3) and wheat straw hydrolysate experiments (Case 4) are presented in Fig. 1a–d. Glucose is consumed approx. two times faster when used as sole substrate (Case 1) than in the sugar mixtures (Cases 3 and 4). Xylose, on the other hand, is consumed approx. two times slower when used as sole substrate and is completely consumed after approx. 60 h compared to around 20 h when co-fermented with other sugars (Cases 3 and 4; Fig. 1c, d). The highest production rate of acetate and hydrogen occurred around 20 h both in the sugar mixture and in the wheat straw hydrolysate fermentations. Lactate was formed just after 20 (Case 3) and 30 h (Case 4) reaching in total 0.015 and 0.014 cmol/L, respectively.

The calculated lag phases differed for each experiment. The lag phases of the sugar mixture experiments ranged from 9 to 11 h, whereas the lag phase of the wheat straw hydrolysate experiment was 4 h. This observation could be correlated to the richer nutrient content of wheat straw than the defined sugar mixture medium. A similar observation was found by Pawar et al. [27]. The lag phase with glucose alone was 4 h, but there was no lag phase with xylose alone. It is worth noticing though that it took more effort to initiate growth on xylose than on glucose as two out of four replicates failed, where none of the other experiments (Cases 1, 3, and 4) failed. This is due to that precautions are needed to start a culture on xylose in the absence of yeast extract, such as no sparging for several hours.

**Fig. 2:**
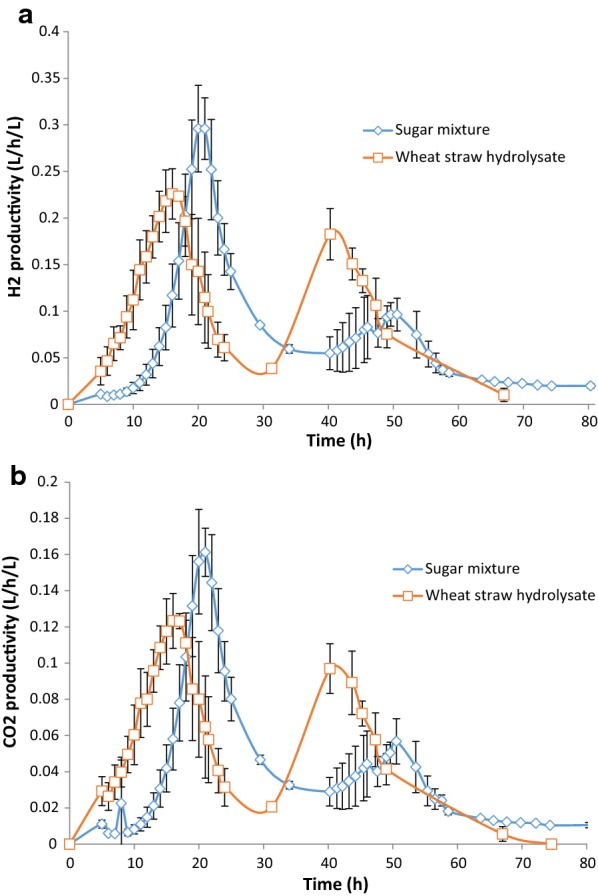
**a** Hydrogen productivity and **b** CO_2_ productivity in Cases 3 and 4, sugar mixture fermentation, and wheat straw hydrolysate fermentation, respectively

The profiles of the mixed sugars indicate a biphasic growth, where the uptake of glucose decreased after xylose was depleted, but then increased again (Fig. 1c, d). The two-phased sugar uptake was more pronounced in the wheat straw hydrolysate fermentations. The behavior can be further illustrated by the hydrogen productivity and CO_2_ productivity (Fig. 2a, b). This observation has, to our knowledge, not been reported for *Caldicellulosiruptor* previously, although the transcriptomics of multiple sugar uptake have been extensively studied [13, 14]. One possible reason for this could be that many multi-sugar experimental studies on this genus have been performed on a yeast extract-supplemented medium [3]. Because yeast extract itself partly supports growth [20], it possibly masks biphasic behavior. Moreover, the initial ratio of pentose/hexose sugars was higher in those studies [14] than in the WSH used in this study. Thus, after xylose was consumed, the culture adapted to a hexose-only medium, which initiated a second phase of growth.

The emerging pattern resembles a diauxic growth behavior, which was first described by Monod [17], and is characterized by two growth phases often separated with a lag period. This normally occurs in the presence of two carbon sources, where the preferred one is consumed first by the microorganism followed by the second after a lag period [28–30]. However, in the case of *C. saccharolyticus,* both pentose and hexose sugars are consumed simultaneously, albeit with a slight preference for the former. When the pentose sugars are depleted hexose consumption continues, but in Case 4 that happened with an increased rate (Table 8).

To quantify this behavior and investigate whether the theory of diauxic growth could be used to explain the observations, a kinetic model was developed consisting of two phases. In the phase I, glucose was consumed simultaneously with xylose and arabinose. Van de Werken et al. [13] concluded that growth on glucose and xylose mixtures as well as growth on the individual sugars all trigger transcription of the genes encoding a xylose-specific ABC transport system. This supports our hypothesis that glucose, xylose, and arabinose were initially transported by the same uptake system. However, when xylose was depleted, phase II starts with a new uptake system being expressed that had a higher affinity for glucose, transporting glucose at an altered rate. It is relevant to observe, however, that diauxic growth behavior is generally considered to be related to PTS systems [31–33]. However, according to current knowledge, *C. saccharolyticus* only possesses ABC transport systems [13, 14]. Still, it has been described that other transport systems can generate this diauxic growth profile. For example, in *Streptomyces coelicolor* and related species, the genes involved in carbon catabolite repression are PTS independent, and instead, glucose kinase is the main controlling enzyme [33].

### Determination of conversion yields

The calculated conversion yields from the batch experiments differ from the stoichiometric yields (Table 6). To begin with, the single sugar fermentations the calculated yields are lower than the corresponding stoichiometric yields. This is in contrast to the yields calculated for the sugar mixture experiments, except for *Y*_ac_ that was slightly lower. The lower yield for acetate could be due to that part of the acetate, or rather acetyl-CoA, which is used as a building block for cell mass production [34].

**Table 6:** Calculated carbon and redox balances plus the calculated yields of the four different experiments and their corresponding stoichiometric yields

	*Y*_*X*_ (cmol/cmol)	*Y*_ac_ (cmol/cmol)	$Y_{{{\text{H}}_{2} }}$ (mol/cmol)	$Y_{{{\text{CO}}_{2} }}$ (cmol/cmol)	Carbon balance	Redox balance
	Yield, biomass formation from sugar	Yield, acetate formation from sugar	Yield, hydrogen formation from sugar	Yield, carbon dioxide formation from sugar	(%)	(%)
Glucose experiments (Case 1)	0.20	0.51	0.45	0.30	82	87
Xylose experiments (Case 2)	0.12	0.50	0.47	0.31	80	81
Sugar mix experiments (Case 3)	0.21	0.62	0.53	0.38	90	100
Wheat straw hydrolysate experiments (Case 4)	0.18	0.68	0.67	0.44	107	90
Stoichiometrically	–	0.67	0.67	0.33	–	–

The carbon balances attained in the model were 90 and 102% with start data from the sugar mixture experiments and the WSH experiments, respectively, which are equal or close to the values calculated from the experimental data, 90 and 107%, respectively, Table 6. The higher values in the carbon balance, i.e., > 100%, for the WSH fermentations, could be due to that other carbon sources may be present, such as oligosaccharides, that are also converted to products giving a higher carbon and electron output.

### Sensitivity analysis

Dynamic simulations using benchmark parameter values [15] showed discrepancies between the experimental results and the model predictions. To further improve the dynamic simulations, a sensitivity analysis was conducted to determine the most important parameters. This was done with start values both from the sugar mixture fermentations as well as from the wheat straw hydrolysate fermentations. The change, *δ*, in the parameter value was set to 1% as in [35].

The sensitivity analysis allowed ranking of the parameters, which was useful for the model calibration. The most sensitive parameters, i.e., with a sensitivity value of > 1%, in regard to each of the state variables are listed in Table 7. The state variables that were affected the most by a change in parameter value were Glu and Xyl. The sensitivities of the other parameters for the different state variables were less than 1%.

**Table 7:** Most sensitive parameters, i.e., sensitivity value > 1%, listed in descending order for each state variable that was evaluated

State variable	Case 3 Sugar mixture	Case 4 Wheat straw hydrolysate
Glu	*k*_m,2_, *α*, *k*_m_, *r*_cd_, $k_{\text{L}} a_{{{\text{H}}_{2} }}$, *K*_s,glu,2_	$k_{\text{L}} a_{{{\text{H}}_{2} }}$, *α*, *k*_m,2_, *r*_cd_, *k*_m_, *K*_s,glu,2_
Xyl	$k_{\text{L}} a_{{{\text{H}}_{2} }}$, *k*_m_, *K*_s,ara_, *K*_s,xyl_	$k_{\text{L}} a_{{{\text{H}}_{2} }}$, *k*_m_, *K*_s,xyl_, *K*_s,ara_, *K*_s,*E*2_
Ara	*K*_s,ara_, *k*_m_, $k_{\text{L}} a_{{{\text{H}}_{2} }}$	*K*_s,ara_
Ac	–	–
*X*	–	–
H_2_	–	*k*_m,2_, α

### Parameter calibration

The sensitivity analysis served as a basis for the parameter calibration. The model was calibrated with data from the four different batch experiments, Cases 1–4. Start values of the state variables were taken from the experimental data (Table 1), and initial parameter values, i.e., benchmark values, were taken from the literature [15] or guesstimated, e.g., by manually fitting the curves of the data points. The calibrated parameters together with a confidence interval of 95% are given in Table 8. Some of the parameters were graphically calibrated and, therefore, are without a confidence interval. The simulations with start data from the single glucose and xylose fermentations were carried out without the diauxic-like growth additions; thus, only phase I was applied.

**Table 8:** Parameters calibrated to experimental data

Parameter	Benchmark value derived from [15]	Case 1	Case 2	Case 3	Case 4
		Glucose simulation	Xylose simulation	Sugar mixture simulation	Wheat straw hydrolysate simulation
*k*_m_, maximal uptake rate (h^−1^)	0.35	–	1.58 (± 0.042)	0.54 (± 0.012)	0.44 (± 0.023)
*k*_m,2_, maximal uptake rate when xylose = 0 (h^−1^)	0.35	2.4 (± 0.15)	–	0.54 (± 0.018)	1.26 (± 0.11)
*K*_s,glu_, affinity constant, glucose (cmol/L)	0.00029	0.01^a^	–	0.01^a^	0.18 (± 0.043)^b^
*K*_s,glu,2_, affinity constant 2, glucose (cmol/L)	–	–	–	0.01^a^	0.01^a^
*K*_s,xyl_, affinity constant, xylose (cmol/L)	–	–	0.0002^a^	0.0002^a^	0.0002^a^
*K*_s,ara_, affinity constant, arabinose (cmol/L)	–	–	–	0.026 (± 0.004)	0.034 (± 0.0077)
*K*_s,*E*2_, affinity constant enzyme, *E*2 (cmol/L)	–	–	–	0.001^a^	0.001^a^
*α*, enzyme synthesis rate (h^−1^)	–	–	–	0.6^a^	0.64 (± 0.085)
*n*, Hill coefficient	–	–	–	2^a^	2^a^
*r*_cd_, cell death rate (h^−1^)	0.014	0.0027^a^	0.0027^a^	0.027^a^	0.027 (± 0.0039)
$k_{\text{L}} a_{{{\text{H}}_{2} }} ,$ volumetric mass transfer coefficient for hydrogen (h^−1^)	0.26	0.44^a^	0.44^a^	0.44 (± 0.085)	0.44^a^
$$ Y_{{{\text{H}}_{2} }} , yield, hydrogen formation from sugar $$		n.c.	n.c.	0.58	n.c.

Confidence interval 95% (CI, 95%) is given for those parameters which have been fitted numerically

*n.c.* not calibrated, but the values calculated from the experimental data were used (Table 6)

^a^ Graphically calibrated

^b^ This value possibly also includes an inhibition factor I

The *k*_m_ values for Cases 3 and 4 describe the maximal simultaneous uptake rates of glucose, xylose, and arabinose (Table 8), and they are modeled with the same value for all the sugars in phase I. However, the *K*_s_ values for glucose in phase I, *K*_s,glu_, are higher than the *K*_s_ values for xylose, *K*_s,xyl_, which indicates a lower affinity for glucose in phase I, since xylose is present and preferred. Moreover, *K*_s,glu_ in Case 4 is 18 times higher compared to *K*_s,glu,2_ and compared to *K*_s,glu_ in Case 3. One explanation is the greater affinity for xylose in phase I and another possible explanation is that *K*_s,glu_ in Case 4 also includes an inhibition term due to the characteristics of the wheat straw hydrolysate media, e.g., Eq. 11:

11 $$ K_{\text{s,glu}} = K_{\text{s,glu, real}} \cdot I, $$

where *I* represents a competitive inhibition, Eq. 12:

12 $$ I = 1 + \frac{{S_{I} }}{{K_{I} }} $$

with *S*_*I*_ the concentration of the inhibitor and *K*_*I*_ the inhibition parameter. This is possibly due to unknown inhibiting compounds in the wheat straw hydrolysate or other factors that inhibit glucose uptake in phase I in Case 4. The reason behind the competitive inhibition has not been identified, but we hypothesize the presence of oligosaccharides that might be preferably taken up instead of glucose. However, these sugars were not quantified in the HPLC analysis of WSH.

The *k*_m,2_ value for Case 4 is 50% lower than the corresponding value for the glucose uptake rate in Case 1. One explanation for this is that the enzymes involved in the sugar uptake in Case 4 take some time to be synthesized making glucose consumption slower in the WSH compared to the single glucose fermentation. Again, the presence of inhibiting compounds or competitive oligosaccharides could further slow down the glucose uptake rate.

Furthermore, the results show that on single sugars and mineral medium, glucose uptake is approximately 35% faster than xylose uptake (Table 8). Moreover, growth of *C. saccharolyticus* on glucose is approx. 40% faster than on xylose (Table 9). This outcome contradicts the previous results on these two sugars in media supplemented with yeast extract (YE), where growth is faster on xylose than on glucose [13, 14]. An explanation for this observation could be that *C. saccharolyticus* needs other sugars (present in YE) to grow optimal on xylose. Indeed, when both sugars are present the growth on xylose is stimulated by the co-uptake of glucose. The stoichiometric relationship of glucose-to-xylose uptake rate *ρ*(Glucose):*ρ*(Xylose) was affected by the media used and is approximately 0.7 and 0.3 in phase I for growth on defined sugar mixture and wheat straw medium, respectively (data used from Fig. 1). Until xylose is depleted, the total glucose, xylose, and arabinose conversion rates, i.e., 0.54·3 h^−1^, are similar to that of xylose conversion in the absence of glucose, i.e., 1.58 h^−1^. This observation is supported by other studies with *C. saccharolyticus* using different sugar mixtures both with and without YE, e.g. in Willquist [36]. Xylose uptake increases if a small concentration of glucose is present or if either the fermentor is sparged with CO_2_ instead of N_2_ gas or closed, to allow buildup of HCO_3_^−^ in the reactor.

**Table 9:** Maximal specific growth rates, *µ*_max_, calculated from *k*_m_, *k*_m,2,_ and *Y*_*x*_ values

Maximal specific growth rate (*µ*_max_, h^−1^)	Phase I	Phase II
Glucose (Case 1)	0.22	–
Xylose (Case 2)	0.13	–
Sugar mixture (Case 3)	0.33	0.11
Wheat straw hydrolysate (Case 4)	0.24	0.23

### Model prediction

**Fig. 3:**
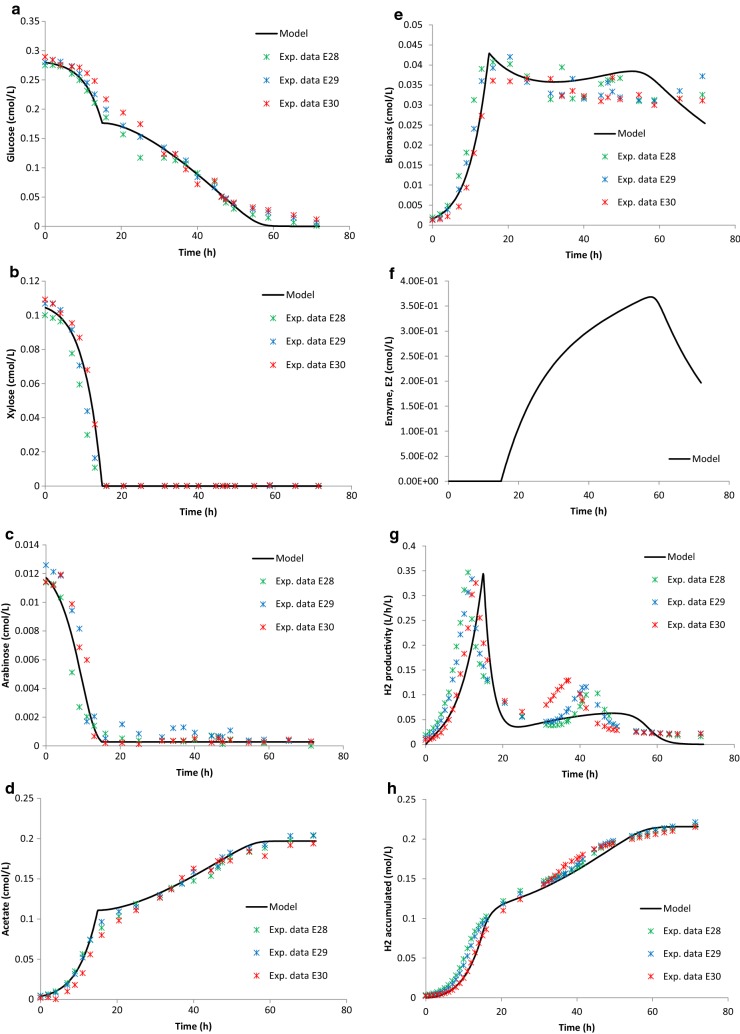
Sugar mixture experimental data and model simulation. **a** Glucose (cmol/L) data and model; **b** xylose data and model (cmol/L); **c** arabinose (cmol/L) data and model; **d** acetate (cmol/L) data and model; **e** biomass (cmol/L) data and model; **f** enzyme, *E*2 (cmol/L) data and model; **g** hydrogen productivity (L/h/L) data and model; and **h** hydrogen accumulated (mol/L) data and model. *Exp. data E28* experimental data E28, *Exp. data E29* experimental data E29, and *Exp. data E30* experimental data E30

**Fig. 4:**
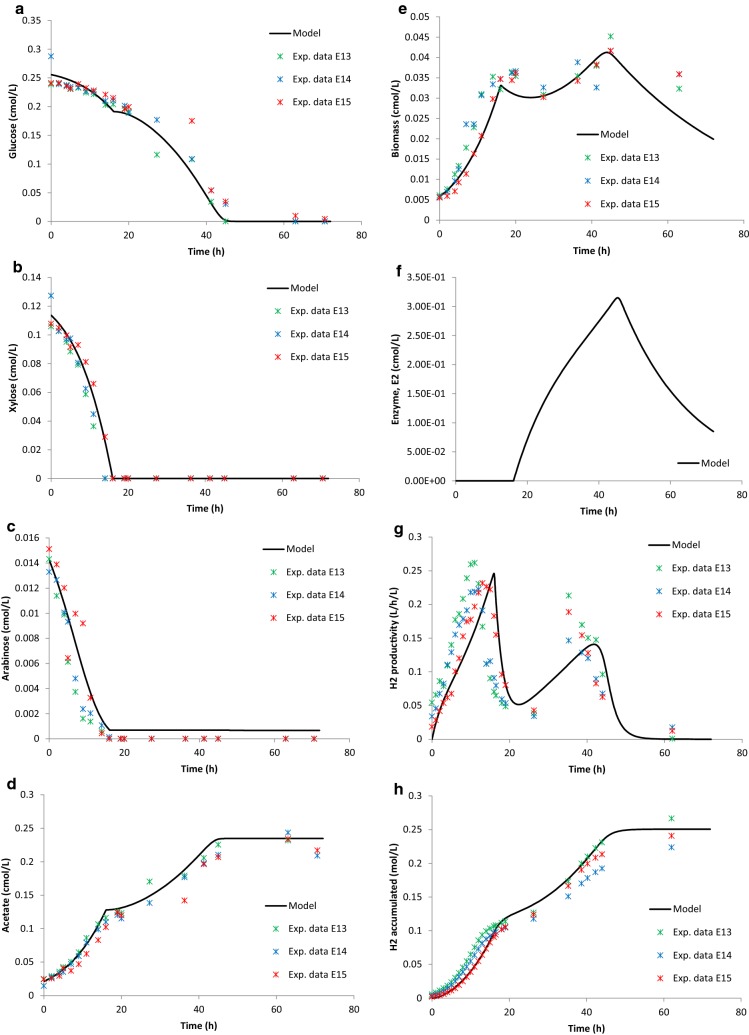
Wheat straw hydrolysate experimental data and model simulation. **a** Glucose (cmol/L) data and model; **b** xylose data and model (cmol/L); **c** arabinose (cmol/L) data and model; **d** acetate (cmol/L) data and model; **e** biomass (cmol/L) data and model; **f** enzyme, *E*2 (cmol/L) data and model; **g** hydrogen productivity (L/h/L) data and model; and **h** hydrogen accumulated (mol/L) data and model. *Exp. data E13* experimental data E13, *Exp. data E14* experimental data E14 and *Exp. data E15* experimental data E15

Comparison between the model and experimental results for the combined sugars is depicted in Table 10, and Figs. 3 and 4. The results show that a diauxic-like behavior model simulates well the experimental data of *C. saccharolyticus* when grown on mixtures of pentose and hexose sugars. Without the addition of a second enzyme equation as well as cybernetic variables controlling the upregulation of the enzyme, the experimental data could not be simulated.

**Table 10:** *R*^2^ values to describe the fit between experimental data and model simulation

State variable	Glucose (Case 1)	Xylose (Case 2)	Sugar mixture (Case 3)	Wheat straw hydrolysate (Case 4)
Glu	0.96	–	0.99	0.97
Xyl	–	0.98	0.99	0.99
Ara	–	–	0.99	0.95
*X*	0.46	0.86	0.92	0.90
Ac	0.91	0.99	0.99	0.99
H_2_ accumulated	0.74	0.99	0.99	0.98

Table 10 shows the fitting between the experimental data and the model simulation displaying the regression analysis values. It is clear that the model is well able to describe the consumption of the different sugars as well as biomass growth, acetate formation, and accumulation of hydrogen in Cases 3 and 4. The model, without the diauxic-like additions, was better at describing the individual xylose fermentations (Case 2), rather than the individual glucose fermentations (Case 1) when it comes to biomass growth and hydrogen production (Table 10).

The model only predicts a small second peak in hydrogen productivity compared to the data of the defined sugar mixture fermentations (Fig. 3g). However, the model succeeds in describing the diauxic-like behavior of the hydrogen productivity profile in the wheat straw hydrolysate fermentations (Fig. 4g). The uptake of the three sugars as well as the formation of acetate is well described by the model, both for Cases 3 and 4 (Figs. 3a–d, 4a–d).

According to the simulation, the enzyme (used to describe the diauxic behavior) concentration is very low, close to zero, in the beginning, and when phase I ends, the enzyme synthesis starts and the concentration increases up to a peak, where it begins decreasing just before *t* = 60 h in Case 3 and somewhat earlier in Case 4 (Figs. 3f, 4f). The enzyme synthesis is dependent on the biomass concentration, which is why it follows the behavior of the latter. The two biomass growth phases are clearly displayed in Case 4 and expressed by the model (Fig. 4e), where a first growth phase takes place between 0 and 20 h and a second growth phase between 20 and 45 h. The phenomenon with two growth phases is characteristic for diauxic growth behavior as described in various literatures on the topic [18, 28, 37].

The hydrogen productivity profile, both in Cases 3 and 4, is a bit delayed in the model (Figs. 3g, 4g). This could be due to a slight underestimation of the $k_{\text{L}} a_{{{\text{H}}_{2} }}$ value. The benchmark $k_{\text{L}} a_{{{\text{H}}_{2} }}$ value used, from Ljunggren et al. [15], was later on calibrated against experimental data resulting in a higher value (Table 8). Still, the mass transfer seems to be less efficient in the model not being able to fully describe the experimental data.

## Conclusions

The outcome of this study revealed that in batch mode, *C. saccharolyticus* ferments (un)defined sugar mixtures via different growth phases in a diauxic-like manner. This behavior could be successfully simulated with a kinetic growth model with substrate-based Monod-type kinetics and enzyme synthesis using Hill kinetics together with cybernetic variables to control the upregulation of the enzyme. The model was able to predict the behavior of growth on sugar mixtures both in a defined medium and in wheat straw hydrolysate medium. The model supported the following sequence: xylose is the preferred substrate, but glucose is taken up simultaneously, possibly with the same transporter. After xylose is depleted, glucose is further taken up with a newly induced transporter system, leading to a second hydrogen productivity peak. We further conjecture that this diauxic-like pattern might appear in defined media not containing complex nutrient mixtures, such as yeast extract, as the latter might reduce the edge of the transition point from dominant xylose uptake to dominant glucose uptake by *C. saccharolyticus*. Future studies should aim at investigating how the various uptake mechanisms in *C. saccharolyticus* act and contribute to the phenomena described in this study. In addition, a further developed model, verifying the values of several kinetic parameters, including separate maximal uptake rates for the different sugars in the sugar mixture as well as inhibition functions, would improve the applicability of this model for industrial processes.
